# Influence of Precipitation and Crop Germination on Resource Selection by Mule Deer (*Odocoileus hemionus*) in Southwest Colorado

**DOI:** 10.1038/s41598-017-15482-7

**Published:** 2017-11-09

**Authors:** Emily M. Carrollo, Heather E. Johnson, Justin W. Fischer, Matthew Hammond, Patricia D. Dorsey, Charles Anderson, Kurt C. Vercauteren, W. David Walter

**Affiliations:** 1 Pennsylvania Cooperative Fish and Wildlife Research Unit, The Pennsylvania State University, 433 Forest Resources Building, University Park, Pennsylvania, PA 16802 USA; 2 Colorado Parks and Wildlife, 415 Turner Drive, Durango, CO 81303 USA; 3 United States Department of Agriculture, Animal and Plant Health Inspection Service, Wildlife Services, National Wildlife Research Center, 4101 LaPorte Avenue, Fort Collins, CO 80521 USA; 4 U.S. Geological Survey, Pennsylvania Cooperative Fish and Wildlife Research Unit, 403 Forest Resources Building, The Pennsylvania State University, University Park, Pennsylvania, PA 16802 USA; 5 Present Address: Missouri Department of Conservation, 2901W Truman Boulevard, Jefferson City, MO 65109 USA

## Abstract

Mule deer (*Odocoileus hemionus*) populations in the western United States provide many benefits to local economies but can also cause considerable damage to agriculture, particularly damage to lucrative crops. Limited information exists to understand resource selection of mule deer in response to annual variation in crop rotation and climatic conditions. We tested the hypothesis that mule deer select certain crops, and in particular sunflower, based on annual climatic variability. Our objective was to use movements, estimates of home range, and resource selection analysis to identify resources selected by mule deer. We used annually-derived crop-specific datasets along with Global Positioning System collars to monitor 14 mule deer in an agricultural area near public lands in southwestern Colorado, USA. We estimated home ranges for two winter seasons that ranged between 7.68 and 9.88 km^2^, and for two summer seasons that ranged between 5.51 and 6.24 km^2^. Mule deer selected areas closer to forest and alfalfa for most periods during 2012, but selected areas closer to sunflower in a majority of periods during 2013. Considerable annual variation in climate patterns and precipitation levels appeared to influence selection by mule deer because of variability in crop rotation and success of germination of specific crops.

## Introduction

Mule deer *(Odocoileus hemionus)* are an important game species in the western United States that can also cause large amounts of damage to agriculture crops. For example, damage by a variety of ungulate species, including mule deer, has resulted in as much as US$100 million annually in economic losses^1–3^. State wildlife agencies often have to reimburse farmers for damage caused by deer and other wildlife, which can be expensive for state agencies^3^ and can degrade incentives for landowners to maintain habitat for wildlife^2^. Multiple measures have been implemented to prevent crop damage by deer such as increasing distances between cropland and key foraging areas of deer^4,5^, using exclusionary methods such as fencing and repellents^1^, and rotating or moving crops that are considered to be favored by cervid species away from areas where they are highly depredated^4,6^.

Sunflower depredation by mule deer has become a significant management challenge in southwest Colorado, because it is a lucrative crop that can experience high rates of cervid damage^1^. Sunflowers are an important crop in the biofuel industry, and high depredation rates by local mule deer populations can cause fields to be completely decimated. Sunflowers in this region are grown on a rotational basis every 3–4 years with other crops that do not receive as much damage by mule deer. Damage by mule deer in Colorado during the 2011/2012 fiscal year resulted in the second highest damage claim ever paid by Colorado Parks and Wildlife at US$292,315^7^. Thus, both farmers and wildlife agencies continuously lose money due to the combination of high depredation rates of various high value crops like sunflower, and the limited information on mule deer populations and their resource selection in this area.

In southwest Colorado several methods (i.e. electric fence, winged fence, polypropylene fence, and a repellent) have recently been explored to prevent crop damage^1^, but very little information exists about resource selection by mule deer in this area. Few studies have documented resource selection of mule deer^8–11^, and even fewer have documented landscape-level selection of crops directly using Global Positioning System (GPS) datasets^12,13^. The few studies that have documented resource selection of mule deer suggested habitat use can be influenced by forage availability, cover, anthropogenic disturbance and water availability^14–16^. Mule deer in western Nebraska selected forested habitats that were near croplands during various seasons^13^. High quality forage and proximate cover also influence habitat use in arid environments of southern California^8^. Although studies have identified resource selection of mule deer, direct observation of changing habitat use trends is limited using GPS-monitored deer^8^.

Resource selection studies typically use static National Land Cover Database layers prepared every 3–5 years rather than annually-derived datasets. Static data layers do not represent annual changes in vegetation potentially lost by urban development and are not specific to crop types that can be rotated on an annual basis. Using annually created layers that match the time period within which GPS datasets were collected is necessary to document fine scale resource selection and would more accurately identify annual changes in crop rotation of agricultural practices. Our objective was to identify resource selection to assess preferences of crops by mule deer, and in particular if there is a preference for sunflower in a predominately agricultural area of arid southwestern Colorado. Our specific objectives were to: (1) estimate home ranges of mule deer in an agricultural area to assess seasonal differences in size, (2) identify general resource selection by mule deer in this area where currently no available data exists, and (3) determine if variation in selection of crops by mule deer in years of varying precipitation levels could be documented using crop-specific data derived annually for this area.

## Study Area

**Figure 1:**
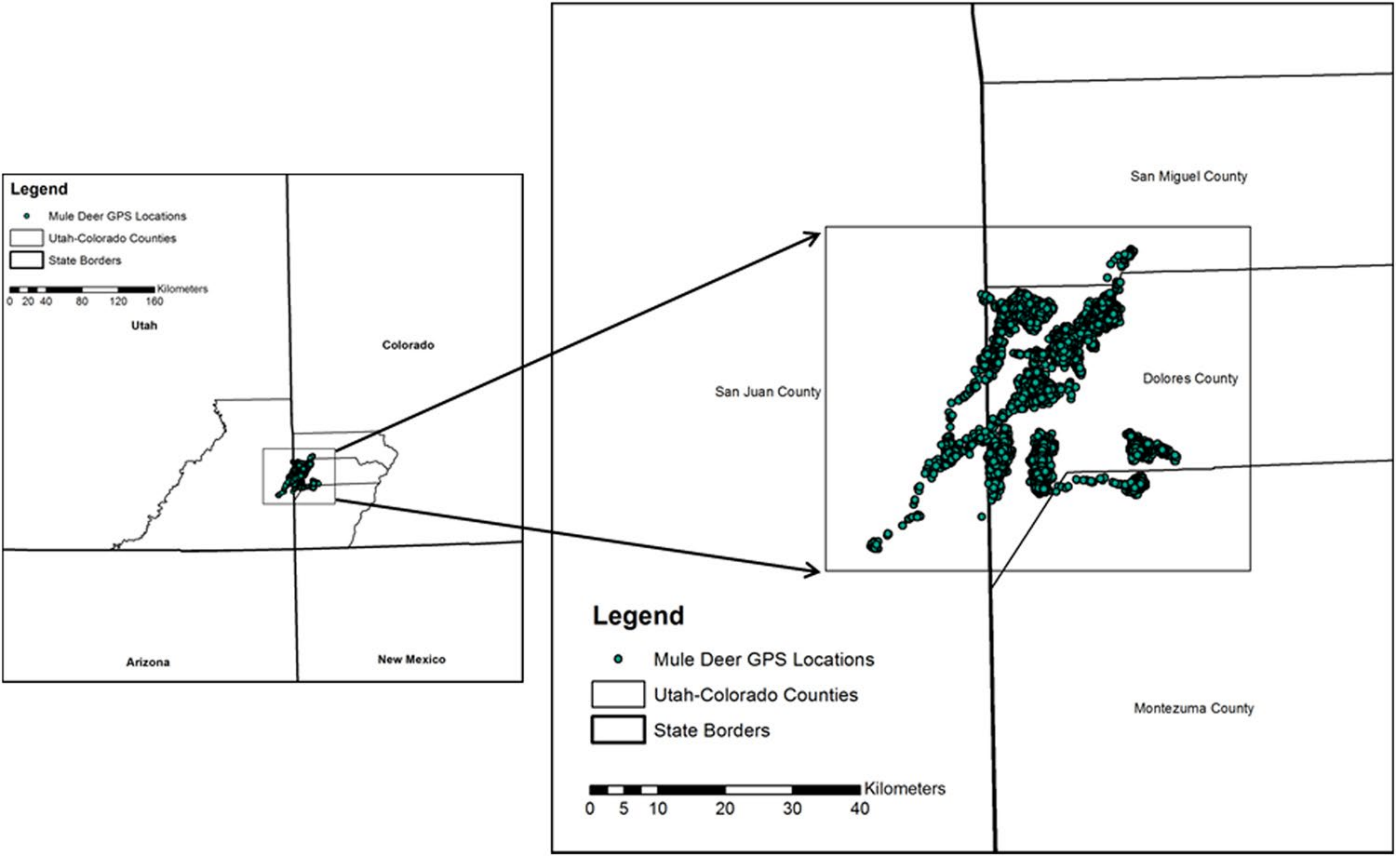
Location of mule deer equipped with Global Positioning System collars in southwestern Colorado and southeastern Utah with state and county borders. Generated with ArcMap 10.2, www.esri.com.

**Figure 2:**
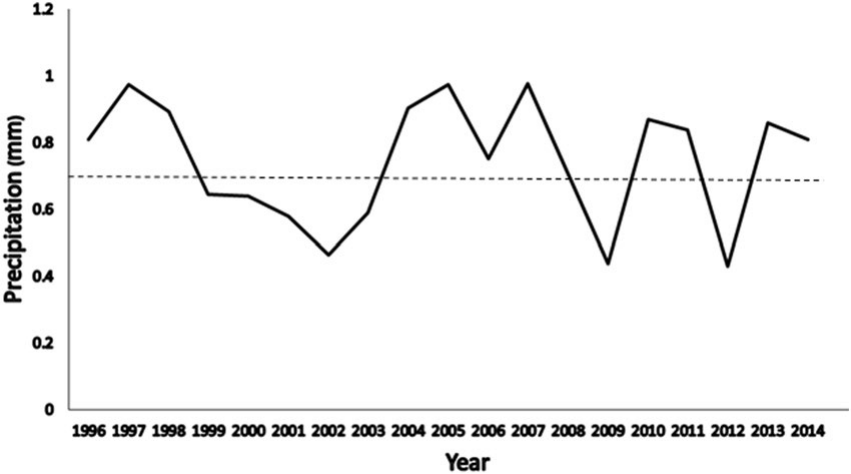
Mean daily precipitation (mm) from 1996 to 2014 in southwestern Colorado and southeastern Utah in areas used by mule deer. Dashed line reflects mean average daily precipitation over the entire time period reflected in the figure.

The study area was approximately 1596 km^2^ in size, and was in the vicinity of Dolores County, Colorado, USA (37.736°N, −108.923°E) (Fig. 1). This area was a mix of public and privately owned properties. Private property was primarily agriculture and public property was primarily native habitat managed by the Department of the Interior’s Bureau of Land Management (federal lands) and Colorado Parks and Wildlife (state lands). The elevation in the study area ranged from 1,981 m to 2,590 m. The local vegetation was characterized as mountain shrub and woodlands that are interspersed with irrigated and dryland agriculture^1^. The primary native vegetation consisted of serviceberry (*Amelanchier alnifolia*), bitterbrush (*Purshia tridentata*), mountain mahogany (*Cercocarpus montanus*), squaw apple (*Peraphyllum ramosissimum*), black sagebrush (*Artemisia nova*), pinyon pine (*Pinus edulis*), and juniper (*Juniperus osteosperma*). Mean total annual precipitation was 26.7 cm between 1996 and 2014, which was received mostly during late summer monsoon rains and during the winter as snowfall (Weather Station DVCO1, Colorado Agricultural Meteorological Network 2013). Yearly precipitation totals for our three study years were 30.6 cm for 2011, 15.7 cm for 2012, and 31.3 cm for 2013 respectively (Fig. 2).

## Results

### Movements and Home Range

Fourteen of the twenty mule deer equipped with GPS collars were available for our analysis. Three collars failed to release on the scheduled drop date, and three deer perished within three months of being collared so were excluded from the study. We collected a total of 56,811 GPS locations for use in our analysis after removing errors in GPS data due to potential outliers caused by poor GPS fixes (i.e., 2-dimensional satellite fixes). We had a mean 3-dimensional fix rate of 97%, and a mean of 4,057 locations per deer. Mean daily movement distance across all deer in our study was 628 m (±262 m SD), which is the distance we used for the radius of our buffered circles. The mean winter home range for 2012 and 2013 was 9.88 km^2^ (±3.87 km^2^) and 7.68 km^2^ (±2.75 km^2^), respectively. The mean summer home range for 2012 and 2013 was 6.23 km^2^ (±3.20 km^2^) and 5.51 km^2^ (±3.22 km^2^), respectively.

### Resource Selection

**Figure 3:**
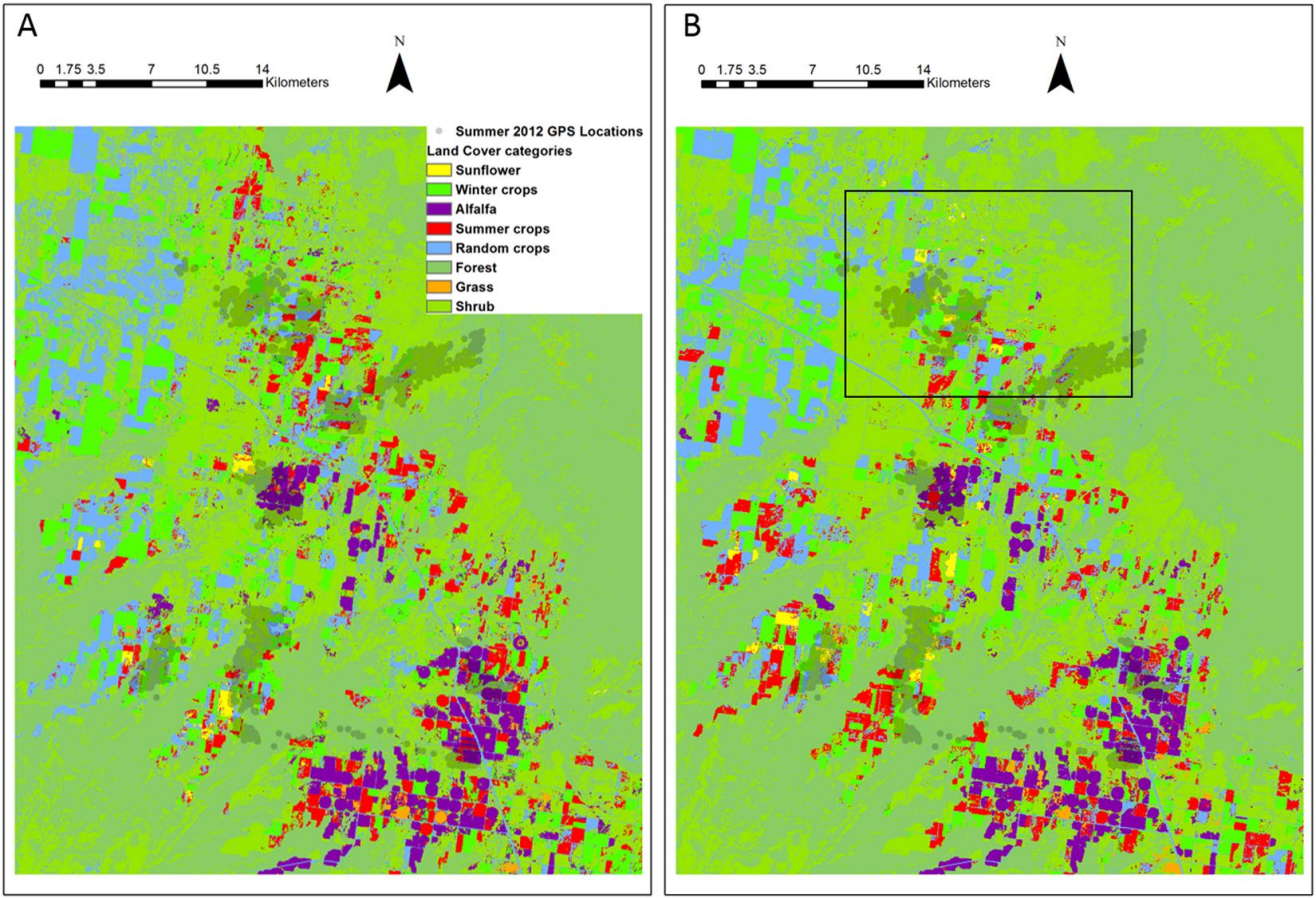
Location of sunflower (yellow), alfalfa (purple), and forest (green) in an area occupied by mule deer equipped with Global Positioning System collars during the summer months in southwestern Colorado and southeastern Utah in (**A**) 2012 and (**B**) 2013. Generated with ArcMap 10.2, www.esri.com. Note the area designated in the box in (**B**) summer 2013 reflects sunflower not present in (**A**) summer 2012 due to lack of adequate precipitation for sunflower to germinate.

Models with the most support indicated that distance to forest influenced nocturnal and diurnal resource selection during both seasons regardless of year (Tables 1 and 2). Mule deer selected areas closer to forests for all seasons and diel periods as indicated by negative coefficients and confidence intervals that did not overlap zero (Tables 3 and 4). Furthermore, mule deer selected areas closer to alfalfa during all season and diel periods except for both diel periods during summer 2013 (Tables 1 and 2). During the summer 2012 season and during both winter seasons resource selection of mule deer was driven mostly by minimizing distance to forest and alfalfa along with some combinations of the other covariates (Tables 1 and 2; Fig. 3).

**Table 1:** Top models using Akaike’s Information Criteria (AICc) adjusted for small sample size with delta AICc < 2.0 for nocturnal locations during summer and winter 2012 and 2013 using mixed-effects logistic regression for mule deer in southwest Colorado.

Model Terms	df	AICc	∆AICc	Weight
*Winter 2012*				
Alfalfa + Roads + Shrub + ---- + Forest	6	66525.4	0.00	0.683
Alfalfa + Roads + Shrub + Sunflower + Forest	7	66527.0	1.56	0.312
*Summer 2012*				
Alfalfa + Roads + ---- + Sunflower + Forest	5	15040.4	0.00	0.335
Alfalfa + Roads + Shrub + Sunflower + Forest	4	15040.4	0.00	0.241
Alfalfa + Roads + ---- + ---- + Forest	5	15041.2	0.82	0.228
Alfalfa + Roads + Shrub + ---- + Forest	6	15041.9	1.48	0.174
*Winter 2013*				
Alfalfa + Road + Shrub + Sunflower + Forest	7	47363.6	0.00	0.755
Alfalfa + Road + Shrub + ---- + Forest	6	47366.4	2.74	0.192
*Summer 2013*				
Alfalfa + ---- + ---- + Sunflower + Forest	5	15040.4	0.00	0.230
---- + ---- + ---- + Sunflower + Forest	4	15040.4	0.00	0.230
---- + Roads + ---- + Sunflower + Forest	5	15041.2	0.82	0.153
Alfalfa + Roads + ---- + Sunflower + Forest	6	15041.9	1.48	0.110
Alfalfa + ---- + Shrub + Sunflower + Forest	6	15042.3	1.89	0.089
---- + ---- + Shrub + Sunflower + Forest	5	15042.4	1.98	0.086

Fixed effects included distance to alfalfa, roads, shrub, sunflower, and forest and random effects were the individual animals.

**Table 2:** Top models using Akaike’s Information Criteria (AICc) adjusted for small sample size with delta AICc < 2.0 for diurnal locations during summer and winter 2012 and 2013 using mixed-effects logistic regression for mule deer in southwest Colorado.

Model Terms	df	AICc	∆AICc	Weight
*Winter 2012*				
Alfalfa + Roads + Shrub + Sunflower + Forest	7	55196.4	0.00	0.566
Alfalfa + Roads + Shrub + ---- + Forest	6	55198.0	1.54	0.262
*Summer 2012*				
Alfalfa + Roads + Shrub + ---- + Forest	6	31204.0	0.00	0.377
Alfalfa + Roads + Shrub + Sunflower + Forest	7	31024.6	0.61	0.277
Alfalfa + ---- + Shrub + ---- + Forest	5	31025.9	1.97	0.141
*Winter 2013*				
Alfalfa + ---- + Shrub + Sunflower + Forest	6	40409.3	0.00	0.623
Alfalfa + Road + Shrub + Sunflower + Forest	7	40411.3	1.98	0.232
*Summer 2013*				
---- + ---- + ---- + Sunflower + Forest	4	24478.6	0.00	0.298
Alfalfa + ---- + ---- + Sunflower + Forest	5	24479.7	1.10	0.172
---- + ---- + Shrub + Sunflower + Forest	5	24480.1	1.52	0.139
---- + Roads + ---- + Sunflower + Forest	5	24480.5	1.89	0.116

Fixed effects included distance to alfalfa, roads, shrub, sunflower, and forest and random effects were the individual animals.

**Table 3:** Parameters, model coefficients (Estimates), standard error (SE), and 95% Confidence Intervals (CI) for summer and winter 2012 and 2013 from the top model during nocturnal hours for selection of crops by mule deer in southwestern Colorado.

Parameter	Estimates	SE	CI
*Winter 2012*			
Intercept	−1.651	0.010	−1.671 to −1.6307
Forest	−0.335	0.013	−0.361 to −0.310
Alfalfa	−0.090	0.012	−0.1104 to −0.0696
Road	0.035	0.009	0.0156 to 0.0537
Shrub	−0.113	0.012	−0.1364 to −0.0902
*Summer 2012*			
Intercept	−1.648	0.018	−1.6837 to −1.6116
Sunflower	0.046	0.027	−0.0079 to 0.0970
Forest	−0.349	0.024	−0.3932 to −0.3038
Alfalfa	−0.102	0.028	−0.1547 to −0.0496
Road	0.061	0.018	0.0255 to 0.0971
*Winter 2013*			
Intercept	−1.653	0.012	−1.6771 to −1.6296
Sunflower	0.034	0.016	0.0034 to 0.0646
Forest	−0.352	0.016	−0.3832 to −0.3213
Alfalfa	−0.123	0.018	−0.1538 to −0.0898
Road	0.034	0.012	0.0109 to 0.0567
Shrub	−0.053	0.015	−0.0818 to −0.0242
*Summer 2013*			
Intercept	−1.686	0.022	−1.7297 to −1.6425
Sunflower	−0.078	0.021	−0.1189 to −0.0364
Forest	−0.534	0.031	−0.595 to −0.4732
Alfalfa	−0.030	0.022	−0.0725 to 0.0117

**Table 4:** Parameters, model coefficients (Estimates), standard error (SE), and 95% Confidence Intervals (CI) for summer and winter 2012 and 2013 from the top model during diurnal hours for selection of crops by mule deer in southwestern Colorado.

Parameter	Estimates	SE	CI
*Winter 2012*			
Intercept	−1.636	0.011	−1.6575 to −1.6142
Sunflower	0.029	0.015	−0.0011 to 0.0586
Forest	−0.256	0.014	−0.2829 to −0.2299
Alfalfa	−0.116	0.016	−0.1476 to −0.0853
Road	0.025	0.011	0.0042 to 0.0465
Shrub	−0.095	0.013	−0.1195 to −0.0696
*Summer 2012*			
Intercept	−1.629	0.015	−1.6579 to −1.6005
Forest	−0.264	0.019	−0.3005 to −0.2276
Alfalfa	−0.057	0.015	−0.0871 to −0.0271
Road	0.029	0.015	0.0006 to 0.0574
Shrub	0.039	0.016	0.0069 to 0.0715
*Winter 2013*			
Intercept	−1.644	0.013	−1.6697 to −1.6187
Sunflower	0.042	0.018	0.0071 to 0.0767
Forest	−0.294	0.016	−0.3262 to −0.262
Alfalfa	−0.143	0.019	−0.1797 to −0.1066
Shrub	−0.088	0.016	−0.1189 to −0.0566
*Summer 2013*			
Intercept	−1.639	0.017	−1.6712 to −1.6059
Sunflower	−0.052	0.017	−0.084 to −0.0193
Forest	−0.313	0.021	−0.3532 to −0.2725

Mule deer selected cover (i.e., forest) and alfalfa, a nutritious forage that is available year-round in agricultural areas during summer 2012 but not summer 2013 (Tables 3 and 4). Mule deer selecting areas closer to sunflower during the summer 2013 but not summer 2012 for both diel periods indicated that this crop was most influential to mule deer resource selection along with distance to forest (Tables 1 and 2; Fig. 3). Mule deer selected sunflower during both diel periods for summer 2013 (Tables 3 and 4) and not during 2012 suggested the lack of availability of this preferred crop due to lack of precipitation. Conversely, sunflower influenced selection during both diel periods for winter 2013; however, positive coefficients indicated mule deer avoided sunflower during winter, which is after sunflower was harvested thus not available. All other vegetation covariates varied in inclusion within the top model, but both distance to roads and shrub tended to have more support during the 2012 season and diel periods which coincided with low precipitation levels in the area (Tables 3 and 4). Cross-validated Spearman-rank correlations for each season and period combination indicated good model performance except for the diurnal period during winter 2012 for unknown reasons (*P* = 0.15; Table 5).

**Table 5:** The predictive ability of resource selection models from Tables 1 and 2 using cross-validated Spearman-rank correlations for mule deer in southwestern Colorado.

Set	Winter 2012		Summer 2012		Winter 2013		Summer 2013	
	*r*_*s*_	*P*	*r*_*s*_	*P*	*r*_*s*_	*P*	*r*_*s*_	*P*
*Day*								
1	0.6760	0.1515	0.988	<0.001	0.988	<0.001	0.952	<0.001
2	0.6760	0.1515	0.976	<0.001	0.988	<0.001	0.952	<0.001
3	0.6760	0.1515	0.997	<0.001	0.988	<0.001	0.879	<0.001
4	0.6760	0.1515	1.0	<0.001	0.976	<0.001	0.915	<0.001
5	0.6760	0.1515	1.0	<0.001	0.988	<0.001	0.915	<0.001
*Night*								
1	1.0	<0.001	1.0	<0.001	0.927	<0.001	0.842	<0.001
2	1.0	<0.001	1.0	<0.001	0.964	<0.001	0.879	<0.001
3	0.988	<0.001	1.0	<0.001	0.927	<0.001	0.891	<0.001
4	1.0	<0.001	0.983	<0.001	0.964	<0.001	0.842	<0.001
5	1.0	<0.001	1.0	<0.001	0.927	<0.001	0.891	<0.001

## Discussion

Our research provides the first reports of size of home range and selection of agricultural crops by mule deer in southwestern Colorado. Our use of crop-specific data derived annually from remote sensing technology (Cropland Data Layer managed by the United States Department of Agriculture) provided detailed information for analysis of resource selection not previously possible with static land cover datasets that are typically created in 5–10 year increments (e.g., National Land Cover Database). We identified a change in crop selection for mule deer from alfalfa to sunflower over two subsequent summers in response to changes in precipitation levels, which altered plantings and success of germination of sunflower and results in different levels of damage^1^. Furthermore, our home range analysis provided detailed information for areas occupied by mule deer in this region throughout the year and potential areas that could sustain crop damage in the future.

Our mean seasonal home range estimates were similar to previously reported home ranges^16,17^ although the use of GPS technology compared to very high frequency technology, and differences in estimator differed from other home range studies. Our mean adult female mule deer home ranges during summer (5.51–6.24 km^2^) and winter (7.68–9.88 km^2^) were similar to mean summer and winter home range for resident adult female mule deer in California of 5.99 km^2^ (±1.89 km^2^ SD) and 10.28 km^2^ (±9.44 km^2^ SD), respectively^16^. Kie, *et al*.^17^ documented mean summer and winter size of home range for adult female mule deer also in California of 6.64 km^2^ (±3.75 km^2^ SD) and 11.38 km^2^ (±8.71 km^2^ SD), respectively. Although our study was the first to use GPS technology and movement-based kernel density estimator (MKDE) to estimate size of home range for mule deer, our apparently smaller home ranges during the summer compared to the winter seasons were similar to other studies^16–18^. Larger home ranges during the winter months (although not statistically so in our study) are common for cervids due to lack of forage close to cover, and could also be due to lack of native forage or variety of agricultural crops that are available during the summer season but not the winter seasons.

Distance to forest influenced resource selection regardless of season or time of day as expected for mule deer that are associated with forested areas. This also confirmed that cover is selected by deer that are influenced by human activity^18–20^. Forest cover also likely provides relief from effects of solar radiation and precipitation. For these reasons, association of our deer with forested habitat was expected. Although most forested habitat in this area was associated with areas of low-elevation riparian depressions, mule deer tended to occupy the periphery of these areas nearest agricultural fields rather than in the center of riparian depressions on public land suggesting that along with cover, easily accessible forage is also important.

Sunflower was selected more frequently by mule deer during the 2013 summer season than any other season, and this may have been due to large differences in precipitation levels. Varying precipitation levels during the 2012 and 2013 influenced the planting and germination of certain crops, especially sunflower, in this area and resulted in different levels of sunflower damage^1^. The average annual precipitation for 2012 was about 50% less than the average annual precipitation for 2011 and 2013, and about 41% less than long-term annual average that has been recorded since 1996 (Weather Station DVCO1, Colorado Agricultural Meteorological Network 2013). Johnson *et al*.^1^ noted that spring seasonal precipitation in 2012 was only 30% of the average spring (March–June) precipitation for this region. This time period is crucial for dryland farming in southwest Colorado, and because of the low precipitation many did not have successful sunflower crops in 2012. Thus sunflower, which is not as drought resistant as other crops like alfalfa, was not as available in 2012 to mule deer as it was in 2013^21^. This may have caused mule deer to select for alfalfa in 2012, but revert back to sunflower in 2013 when it was once again available.

We acknowledge the limitations of our study due to low sample size as well as deer occupying similar areas at our study site. Due to costs of additional collars and access to private property by the helicopter capture crew, we were unable to supplement our original sample size although our sample size with GPS technology reflected numerous deer spread across our study area (Fig. 1). We previously documented crop damage of sunflower in this area through use of exclusion fencing and differences in damage during years of low and high precipitation (Johnson *et al*.^1^). Although these areas did not overlap entirely, our limited GPS dataset further supported Johnson *et al*.^1^ that mule deer react to sunflower based on precipitation levels that fluctuate annually as well as proximity to security cover provided by forested habitat. Furthermore, although the influence of water availability was not specifically addressed, future research focusing on human-derived water sources or detailed spatial layers on water availability may offer insight into resource selection during years of low precipitation.

Utilizing crop-specific data derived annually was necessary to achieve our objectives that would not have been possible using static data layers. Appropriate data layers (annually-derived, crop-specific), study design (matched *case-control*), and accounting for variation in sampling frequency among individual animals (random effect) enabled detailed resource selection analysis to achieve study objectives of population-level RSFs^22^. Methods to prevent crop depredation can be expensive and time consuming to implement on an annual basis. The ability to identify areas with agriculture and crop type that need the most protection would greatly reduce the amount of money and time lost by both landowners and wildlife agencies when implementing various crop depredation prevention strategies. To further complicate matters, crop depredation varies annually depending on local precipitation and temperature patterns that we documented through exclusionary fencing and GPS-collared deer in the current study (Johnson *et al*.^1^). This variation in precipitation directly influences crop rotation, timing of germination and sprouting of plants. This is especially true for areas like southwest Colorado that can have large variation in precipitation levels and temperature and thus crop success.

## Methods

### Capture and Monitoring

We captured twenty adult female mule deer in September 2011 using a net-gun fired from a helicopter in areas around agricultural fields that had previously experienced crop damage. Each deer received a Telonics store-on-board GPS collar (Product Model: TGW-4501; Telonics, Mesa, Arizona, USA) programmed to collect locations every three hours for two years. All capture and handling methods were in accordance with protocols approved by the Colorado Parks and Wildlife Animal Care and Use Committee (CDOW IACUC No. 05-2011) and within guidelines of the American Society of Mammalogists^23^.

### Movements and Home Range

We created two seasonal categories related to differences in timing of planting crops and thus variation in germination and sprouting, which influences crop availability and preferences by mule deer determined based on research on crop damage in the area^1^. We delineated these two seasons over the two years of the study resulting in four season/year combinations: (1) *winter 2011* from 1 October 2011 to 31 May 2012, (2) *summer 2012* from 1 June 2012 to 30 September 2012, (3) *winter 2012* from 1 October 2012 to 31 May 2013, and (4) *summer 2013* from 1 June 2013 to 30 September 2013.

We determined daily movement distances for mule deer based on a 3-hour collection schedule (i.e., 6–8 locations) for our GPS collars over a 24-h period. Mean daily movement distances were estimated for each monitored deer and used as the distance an average deer could move during any 24-hour period. We used the 95% MKDE to estimate seasonal home ranges for each deer using a biased random bridge approach^24,25^. Unlike traditional kernel density estimators, MKDE can integrate temporal correlation and maximum time lags between subsequent locations leading to more refined movement vectors thus improving estimates of home range over traditional estimators^24–26^. We did not include habitat in MKDE estimation because we wanted to standardize estimation of home range based on our GPS collection schedule regardless of habitat resulting in liberal estimates of home range although still less liberal than most home range estimators (e.g., reference bandwidth smoothing^26^).

#### Vegetation Covariates

We identified eight vegetation categories and a road variable believed to influence selection by mule deer in the western US^10,13,14,27^. We included roads from the United States Census Tiger/Line ascii files (U.S. Census Bureau, Washington, DC, USA) because roads have been documented to influence resource selection by cervid species in previous studies^14,28^. We used annual crop layers from the Cropland Data Layer (CDL) project that is managed by the United States Department of Agriculture, which utilizes Deimos-1, UK-DMC 2, Landsat TM/ETM+ or Landsat 8, and AWiFS imagery for the production of a 30 m national product (USDA-NASS, Washington, DC, USA). Separate CDL layers were downloaded for each of the three years of our study to most accurately reflect crop rotation for *winter 2011*, *summer 2012*, *winter 2012*, and *summer 2013*. Vegetation categories were reclassified into eight categories that were considered important to resource selection by mule deer. *Summer crops* were defined as crops planted in the spring, grown throughout the summer months, and harvested in early autumn such as dry beans, safflower, triticale, oats, barley, corn, sweet corn, sorghum, and flaxseed. *Winter crops* were defined as crops planted in summer or autumn and grown throughout the winter that included rye, speltz, and winter and spring wheat. *Other crops* were crops or other CDL categories occurring in either winter or summer and occurred infrequently or in limited areas across the study site, and were considered least important to mule deer resource selection such as watermelon, grapes, and barren land. *Alfalfa* because it represented a large portion of the available crops and is known to be consumed routinely by mule deer^27^. *Sunflower* because it was the main crop of interest for this area^1^. *Forest* included all forest types, which were mostly composed of pinyon pine and juniper; this category was considered the main cover variable because of the influence forest cover has on the resource selection of cervid species^13,20^. *Shrub* included shrubs less than 5 meters tall with shrub canopy typically greater than 20% of total vegetation and likely provided cover to mule deer. *Grass* included all grassland dominated by gramminoid or herbaceous vegetation. We created a 30 × 30-m raster for each covariate by determining distance from each cell to each of the nine covariates. We did not include elevation because a majority of forest cover occurred in lower elevations adjacent to agricultural fields and grasslands likely resulting in both forest and low elevation yielding similar influences on resource selection.

### Statistical Analysis

We estimated a population-level resource selection function (RSF) using mixed-effects logistic regression models^29^. We examined a correlation matrix for all covariates before modeling to screen for collinearity and covariates with |*r*| > 0.7 during any one period were excluded in all models. *Grass*, *summer crops*, *winter crops*, and *other crops* were not included in final models because of collinearity, and because they occurred less frequently within our study area (i.e., collectively about 17%) compared to the remaining vegetation categories. Using logistic regression with use–availability data presents some problems because predicted values are not scaled between 0 and 1 and generally do not reflect true probabilities of resource selection. Logistic regression can provide an informative and unbiased method for ranking habitat use, however, and for comparing relative probability of use^30,31^. We used individual mule deer as a random-intercept in our mixed-effects models to address issues associated with autocorrelation and uneven sample sizes of locations between individuals^32^ but did not account for individual-level selection because our intent was to estimate a population-level RSF for the study area to achieve our objectives. Furthermore, we modeled diel (night/day) categories separately because of the influence human activity can have on deer behavior^19,20,33^. We modeled population-level RSFs for each of the eight periods (four seasons, two diel) and chose not to use year or diel as fixed or random effects because we were interested in identifying potential differences in selection of crops and not simply accounting for the effect of year or diel in our data^22^. Our study design also considers that various crops are seasonal so some crops were only available during certain seasons and could only be selected by deer during those seasons (i.e., sunflower selection could only occur during the summer seasons because of its absence during the winter seasons).

We used buffered circles to create random points instead of the entire home range to attain a higher order (fourth order) of selection and specificity as it relates to spatial scale^22,34^. We wanted a higher order of selection to identify specific selection among a suite of unique cover and crop categories for each relocation rather than comparing relocations to the same available locations that could be randomly generated within the home range of an animal (i.e., third-order selection). The radius of our buffered circles was determined from the mean daily distance moved that was determined from our 6–8 locations collected per 24-hour period. We generated five random points within each buffered circle for each used point, and the five random points were considered *available* in our RSF analysis and paired with each *used* location for a matched *case-control* analysis^35^. We included standardized distance to the four vegetation categories and roads in a global model that also included the random effect for each animal. We used second-order bias correction for Akaike’s Information Criterion (AICc^36^;) to select the most parsimonious model among a suite of models for each period of analysis. For each period and day/night combination (*n* = 8), we included all possible combinations of the five variables (*n* = 32) for model selection with AICc. We used package *adehabitatLT* for movement analysis^37^, *adehabitatHR* for MKDE estimation^38^, and *lme4* and *MuMIn* for mixed-effects logistic regression and AICc, respectively, all in R (R Foundation for Statistical Computing, Vienna, Austria).

We performed model validation on our top model with a cross-validated Spearman-rank correlation coefficient (r_s_) for 10 bins across 5 training sets^39^. We used a model training-to-testing ratio of 80:20 for the five random subsets by fitting the top model with all data then using the estimated coefficients to obtain predicted values for both training and withheld datasets.

*A posteriori* analysis of precipitation effects in the area revealed a large difference in the amount of precipitation received during 2012 in comparison to 2013 and the regional yearly average from 1996–2012. Total regional precipitation for 2012 was 15.7 cm, and the total for 2013 was 31.3 cm respectively. The average from 1996–2012 was 26.7 cm, so precipitation for 2012 was about 41% less than the yearly average and was about 50% less than it was in 2013 (Fig. 2).
